# Comprehensive plasma metabolomics analysis of berberine treatment in ulcerative colitis rats by LC-MS/MS

**DOI:** 10.3389/fchem.2024.1518110

**Published:** 2024-12-11

**Authors:** Baodong Feng, Linqi Su, Yang Yang, Renyan Liu, Yu Zhang, Lingyi Xin, Li Wang, Zhiming Yang, Xuemei Wei, Qinhua Chen

**Affiliations:** ^1^ Key Laboratory of TCM Clinical Pharmacy, Shenzhen Baoan Authentic TCM Therapy Hospital, Shenzhen, China; ^2^ School of Pharmaceutical Sciences, Hubei University of Medicine, Shiyan, China; ^3^ Department of Pharmacy, The Seventh Clinical College of Guangzhou University of Chinese Medicine, Guangzhou, China

**Keywords:** ulcerative colitis, plasma metabolomics, berberine, HPLC-MS/MS, dynamic multiple reaction monitoring

## Abstract

**Background:**

Ulcerative colitis (UC) is a chronic inflammatory bowel disease (IBD) influenced by multiple factors. Berberine, an isoquinoline alkaloid derived from the root and bark of *Coptis chinensis* Franch., has shown promise in managing UC, but its underlying mechanisms remain unclear.

**Methods:**

To elucidate the relationship between berberine, ulcerative colitis (UC), and the organism’s metabolome, we established a dextran sulfate sodium (DSS)-induced UC model in rats. Colonic tissue was collected for histopathological examination, while plasma samples were analyzed using liquid chromatography-tandem mass spectrometry (LC-MS/MS) with dynamic Multiple Reaction Monitoring (dMRM). This approach, characterized by its short analysis time of 20 min per sample, excellent reproducibility, and straightforward data processing, allowed for the comprehensive detection of a wide array of metabolites, including amino acids, lipids, and organic acids, many of which are implicated in the pathophysiology of UC.

**Results:**

Our results showed that berberine modulated the metabolic disturbances of 33 compounds in the plasma of UC rats, primarily including amino acids, pyrimidines, organic phosphoric acids, fatty acyls, and organonitrogen compounds. These altered metabolites were associated with various pathways, such as amino acid metabolism, glutathione metabolism, nicotinate and nicotinamide metabolism, taurine and hypotaurine metabolism, pyrimidine metabolism, glyoxylate and dicarboxylate metabolism, and the citrate cycle (TCA cycle). Notably, 3-hydroxyproline, homocysteic acid, *L*-threonine, *L*-lysine, carbamoyl phosphate, *O*-phosphoethanolamine, taurine, leucine, and phosphorylcholine exhibited significant differences between the Treatment and Model groups, with levels reverting to those of the Control group (*p* < 0.001). These findings suggested that these compounds may serve as potential plasma biomarkers for UC.

**Conclusion:**

This study provided valuable insights into the mechanism by which berberine exerted its therapeutic effects on UC through metabolomics. Our results highlighted berberine’s potential to modulate key metabolic pathways and restore the levels of several metabolites, suggesting its utility as a therapeutic agent for UC. These findings underscored the importance of metabolomics in understanding the pathophysiology and treatment of UC.

## 1 Introduction

Dynamic homeostasis is a fundamental characteristic of all living systems, entailing the continuous adaptation of organisms in response to a variety of exogenous stimuli, including pharmacological treatments, dietary changes, and environmental factors (De Oliveira Madeira and Antoneli, 2024). To elucidate the intricate complexity and dynamics of these systems, an integrative and holistic approach known as systems biology—or systeomics—is now widely adopted (Tavassoly et al., 2018). The systeomics approach encompasses several pivotal ‘omics’ sciences: genomics, transcriptomics, proteomics, and metabolomics, which respectively aim to delineate the genome, transcriptome, proteome, and metabolome (Demirhan et al., 2022). Initial advancements in genomics enabled the sequencing of genomes across diverse organisms, catalyzing progress in related systems biology disciplines, such as transcriptomics and proteomics (Grogan and Perry, 2020). These ‘omics’ fields are specifically focused on quantifying mRNA transcription levels (the transcriptome) and protein abundances (the proteome). Further developments in proteomics have propelled the evolution of metabolomics, which aims to quantify low-molecular-weight compounds known as metabolites, the expression products of individual proteins (Schrimpe-Rutledge et al., 2016). Metabolomics, the systematic study of small molecule metabolites within biological systems, relies on a suite of analytical techniques to comprehensively profile the metabolome (Bujak et al., 2015). Key methodologies include nuclear magnetic resonance (NMR) spectroscopy and mass spectrometry (MS), both of which offer unique advantages in detecting and quantifying metabolites (Emwas, 2015). While NMR provides structural information with high reproducibility, MS excels in sensitivity and the ability to detect a broad spectrum of metabolites (Moco, 2022). Among these, ultra-high-performance liquid chromatography (UHPLC) coupled with high-resolution mass spectrometry (HRMS) stands out for its unparalleled accuracy in mass measurement and its capacity to resolve complex mixtures.

UHPLC-HRMS enables the identification of a wide range of metabolites, thereby offering profound insights into metabolic pathways and their alterations under various physiological conditions (Ma et al., 2022). This advanced technology has significantly enhanced our ability to discern subtle biochemical changes, contributing to a deeper understanding of metabolic processes and their implications in health and disease (Chen et al., 2022; Chen et al., 2023; Wang et al., 2024). UHPLC-HRMS has revolutionized the field of metabolomics by enabling comprehensive and sensitive detection of a wide array of metabolites. The untargeted approach provided by UHPLC-HRMS offers a broad overview of the metabolome, facilitating the discovery of novel biomarkers and metabolic pathways (Grasso et al., 2022; Wei et al., 2021). Despite its significant contributions to metabolomics, UHPLC-HRMS was not without limitations. One major drawback was the complexity and time-consuming nature of data analysis, exacerbated by the vast amount of data generated. The high sensitivity of UHPLC-HRMS also led to the detection of numerous background signals and non-specific compounds, complicating the analysis (Chen et al., 2013). Additionally, while UHPLC-HRMS provided broad coverage of the metabolome, its quantitative accuracy and reproducibility for specific metabolites may have been inferior to those of targeted approaches (Huang and Zhou, 2022).

To advance the precision and comprehensiveness of metabolic profiling, researchers have pioneered a novel approach known as pseudotargeted metabolomics. This methodology leveraged the transition from UHPLC-HRMS to triple quadrupole mass spectrometry (TQMS) (Chen et al., 2013). By focusing on specific ion transitions, TQMS significantly enhanced the sensitivity and selectivity of metabolite detection, thereby enriching the depth and accuracy of metabolic profiles (Zheng et al., 2020). This evolution underscored a commitment to achieving higher standards of analytical rigor and reliability in metabolomics studies, setting a precedent for the development of more sophisticated and robust analytical frameworks (Liu et al., 2024). While pseudotargeted metabolomics offered substantial benefits, including the potential to identify a broader spectrum of metabolites without the need for authentic standards, it also presented several challenges. The reliance on advanced and costly instrumentation, such as UHPLC-HRMS, and the necessity for complex methodological adjustments and stringent data handling protocols remained significant hurdles. Additionally, the extensive datasets produced often encompassed a multitude of compounds, many of which may not be directly relevant to the study objectives, potentially diverting attention and resources. Collectively, these factors underscore ongoing challenges in streamlining workflows and improving analytical specificity, thereby motivating continuous efforts to refine pseudotargeted metabolomics techniques for enhanced practical utility and efficiency. Building upon the foundation of pseudotargeted metabolomics (Luo et al., 2015; Yuan et al., 2012; Zheng et al., 2020), the present study has developed a method utilizing the dynamic Multiple Reaction Monitoring (MRM) mode of triple quadrupole mass spectrometry (TQMS) to construct a platform capable of simultaneously detecting hundreds of compounds of interest. This approach eschews the need for expensive instrumentation, demonstrating that a targeted analysis can be effectively achieved using solely the TQMS. The simplicity of data processing is a notable advantage, as it obviates the necessity for chromatographic peak extraction and correction, thereby streamlining the workflow. Consequently, sample analysis can be completed within a remarkably short timeframe of 20 min per sample, offering a swift and efficient alternative to traditional methods.

Ulcerative colitis (UC), a chronic manifestation of inflammatory bowel disease (IBD), poses a considerable challenge to public health, characterized by its incapacitating symptoms and persistent inflammation within the digestive tract (Peyrin-Biroulet et al., 2016; Temby et al., 2023). Among the myriad therapeutic strategies explored for the management of UC, berberine—an isoquinoline alkaloid extracted from the traditional Chinese medicinal plant *Coptis chinensis*—has emerged as a promising candidate due to its demonstrated efficacy (Deng et al., 2024). Despite this, the intricate mechanisms through which berberine mediates its therapeutic benefits have yet to be fully elucidated (Song et al., 2020). Previous research has indicated that berberine can influence diverse metabolic disturbances observed in the feces and urine of UC rats (Liao et al., 2019). Although UC predominantly impacts the gastrointestinal system, its systemic nature is evidenced by its association with a range of extraintestinal manifestations (Fabián and Kamaradová, 2022), such as arthritis (Wang and Tsai, 2023), skin issues, ocular problems and liver disorders (Aloi and Cucchiara, 2009). Given these systemic implications, plasma samples offer a non-invasive window into the organism’s global metabolic landscape, providing indispensable insights into both the pathophysiology of UC and the potential systemic effects of therapeutic interventions (Chen et al., 2019). In this study, we utilized comprehensive metabolomics analysis via LC-MS/MS to characterize the plasma metabolome of ulcerative colitis rats following berberine treatment. This approach aims to unravel the metabolic alterations induced by berberine, offering a deeper understanding of its therapeutic mechanisms in the context of UC.

## 2 Materials and methods

### 2.1 Reagents and materials

Berberine Hydrochloride Tablets (0.1 g/Slice, NO. 2230533) and Mesalazine Enteric coated tablets (0.25 g/Slice, NO. H20103359) were respectively purchased from Northeast Pharmaceutical Group Shenyang NO.1 Pharmaceutical CO., LTD. and Heilongjiang Timehome Pharmaceutical CO., LTD. Acetonitrile (LC grage), methanol and formic acid were purchased from Merck and Co., Inc. The reference standards of clenbuterol (NO. WP23112904, ≥98.00%) and chloramphenlcol (NO. L2213370, ≥98.00%) were respectively purchased Shanghai Yuanye Bio-Technology CO., LTD and Shanghai Aladdin Biochemical Technology CO., LTD. The reference standards of *L*-methionine (NO. 24010251, ≥99.30%), sarcosine (NO. 24070588, ≥99.30%), *L*-valine (NO. 24020502, ≥99.90%), *L*-alanine (NO. 24010392, ≥99.80%), *DL*-2-aminooctanoic acid (M16GB148843, ≥98.00%), *L*-glutamic acid (NO. 231002016, ≥99.70%), *L*-threonine (NO. 24020271, ≥99.90%), *L*-lysine (24020360, ≥97.20%), *L*-histidine (NO. 23120455, ≥99.50%), *L*-pipecolic Acid (NO. 22060947, ≥99.90%), guanine (NO. 24030186, ≥99.90%), niacinamide (24010447, ≥99.80%), *O*-phosphoethanolamine (NO. Q15F8S29252, ≥98.00%), taurine (NO. 24030314, ≥98.50%), spermidine (NO.24010276, ≥99.40%) were purchased from Tanmo Quality Inspection Technology CO., LTD. A Milli-Q system (Arium bagtank 50, Sachsen, DEU) was used to produce deionized water. Carboxymethylcellulose sodium (CMC-Na) was purchased from Labgic Technology CO., LTD (Beijing, China), and Dextran Sulfate Sodium Salt (DSS) was purchased from Yeasen Biotechnology (Shanghai) CO., LTD. (Shanghai, China).

### 2.2 Animal study

All animal experiments were approved by Institutional Animal Care and Use Committee of Shenzhen TOP Biotechnology CO., LTD (TOP-IACUC-2024-0083). Male Sprague–Dawley (SD) rats (190–210 g, 7–8 weeks), provided by Guangdong Medical Laboratory Animal Center, were randomly divided into four groups: Control group (treated with water, n = 6), Model group (treated with 4% DSS, n = 6), Berberine group (treated with 4% DSS and Berberine, n = 6) and Mesalazine group (treated with 4% DSS and Mesalazine, n = 6). DSS-induced colitis in Model group, Berberine group and Mesalazine group were established by oral intake of 4% DSS in drinking water for 7 days, while rats in Control group were treated with water. After DSS administration for 24 h, rats in Berberine group and Mesalazine group were respectively gavaged with 100 mg/kg Berberine and 100 mg/kg Mesalazine (dissolving in 0.5% CMC-Na solution) for the last 7 days, while rats in Control and Model group were gavaged with 0.5% CMC-Na solution with equal volume. During the experiment, the food and water intake were observed daily. The Disease Activity Index (DAI) of the rats was determined by body weight loss, stool consistency and the degree of stool occult blood. The scoring system was shown in Supplementary Table S1. The calculation formula for the Disease Activity Index (DAI) is as follows:

$$DAI=\left(Body\;Weight\;Loss+Stool\;Consistency+Occult\;Blood\right)/3$$

Data collection for the DAI was performed daily. Body weight was measured using an electronic scale, and the percentage weight loss relative to the initial weight was calculated. Stool consistency was assessed visually and scored according to the provided criteria. Occult blood was detected using the benzidine method with a fecal occult blood test kit. The DAI was evaluated on the final day of the experiment to assess overall disease severity. Higher DAI values indicated more severe disease activity, while lower values suggested improvement or remission. At the end of the experiment, all rats were anesthetized by tribromoethanol and plasma was taken through the abdominal aorta after fasted for 12 h. The colons were fixed by 4% paraformaldehyde for 48 h and then embedded in paraffin wax. Five-micron-thick sections were cut and stained with hematoxylin-eosin for histopathological evaluation. Plasma samples were separated by centrifugation at 14000 rpm for 10 min and stored at −80°C until further analysis.

### 2.3 Sample preparation for plasma metabolomics

100 μL of plasma sample was spiked with 20 μL of clenbuterol as internal standard (CL, 5 μg/mL) in positive mode and 20 μL of chloramphenicol (CH, 5 μg/mL) as internal standard in negative mode, followed by vortex mixing for 30 s. Then, 400 μL of precipitate solvents (Acetonitrile: Methanol = 1: 1) was added and vortexed for another 3 min. After centrifugation at 14000 rpm for 30 min, 5 μL aliquot of the supernatant was injected into LC-MS/MS system for further analysis. A quality control (QC) sample was produced by mixing and blending equal volumes (10 µL) of each sample. To ensure system equilibrium at the beginning of the analysis, a QC sample was injected 6 times. Subsequently, to monitor system stability throughout the sample testing, a QC sample was injected every six samples. Additionally, a blank sample was run every eight samples to ensure the absence of carryover and contamination. All samples were tested in randomized order to minimize systematic biases and further validate the robustness of the analytical method. All standards were dissolved in 50% methanol water and finally diluted to 50 ng/mL.

### 2.4 HPLC-MS/MS analysis for plasma metabolomics

The HPLC-MS/MS systems consisted of HPLC separation system (Shimadzu, Kyoto, JPN) and an API 6500+ Qtrap mass spectrometer equipped with an ESI interface (AB Sciex, Framingham, MA, United States). Chromatographic separation was conducted using an ACQUITY UPLC HSS T3 column (2.1 mm × 100 mm, 1.8 μm, Waters Corp., Milford, MA, United States) with column temperature maintained at 40°C. The 0.1% formic acid in water as mobile phase A and 0.1% formic acid in acetonitrile as mobile phase B. The gradient elution program with a flow rate of 0.3 mL/min was used and as follows: 0–2 min, 0% B; 2–14 min, 0%–95% B; 14–16 min, 95% B; 16–20 min, 0% B.

Low-resolution MS and MS/MS analysis were performed on an API 6500+ Qtrap mass spectrometer equipped with an ESI interface (AB Sciex, Framingham, MA, United States). Equipment control was performed using Analyst software ver.1.7.2 (AB Sciex). The data analysis was performed by using SCIEX OS software ver.2.1.6.59781 (AB Sciex). The conditions of the MS detector were set as follows: ion spray voltage, 5.5 kV (Positive mode), −4.5 kV (Negative mode); capillary temperature, 550°C; ion source GS1, 70 psi; ion source GS2, 80 psi; curtain gas, 35 psi. The mass spectrum was respectively recorded in the m/z range of 100–1000 in positive mode and negative mode. The dwell time of each ion pair was 2 m. Nitrogen was used in all cases. The MS/MS experiments were conducted following the methodology reported (Chen et al., 2020), with a critical threshold of approximately 3000 counts set for the intensity of precursor ions to ensure high-quality data acquisition and minimize background noise. The mass spectrometric conditions for various metabolites under the Dynamic MRM mode are provided in Supplementary Table S2.

### 2.5 Data analysis of plasma metabolomics

All data were acquired using Analyst software version 1.7.2 (AB Sciex) and subsequently imported into SCIEX OS software version 2.1.6 (AB Sciex) for peak area integration. Prior to statistical analysis, the raw data were filtered to remove noise and artifacts. Specifically, ions with a signal-to-noise ratio below three were excluded to minimize background interference. The peak areas of the identified compounds were then imported into MetaboAnalyst 6.0 for Pareto scaling normalization. Multivariate statistical analyses (PCA and OPLS-DA) were used to the different groups and determine the altered compounds. OPLS-DA models were validated by confusion matrix, cross validation and CV-ANOVA, while OPLS-DA models were validated by cross validation and CV-ANOVA. The altered compounds between each two groups were screened by variable importance in the projection (VIP) ≥1 from the cross-validated OPLS-DA models. The altered metabolites were analyzed by Metabolomics Pathway Analysis (http://www.metaboanalyst.ca/) and were related to potential pathways. KEGG database (http://www.kegg.jp/) was used to further determine the location and function of these altered metabolites in various metabolic pathways.

## 3 Result

### 3.1 Method validation

**FIGURE 1:**
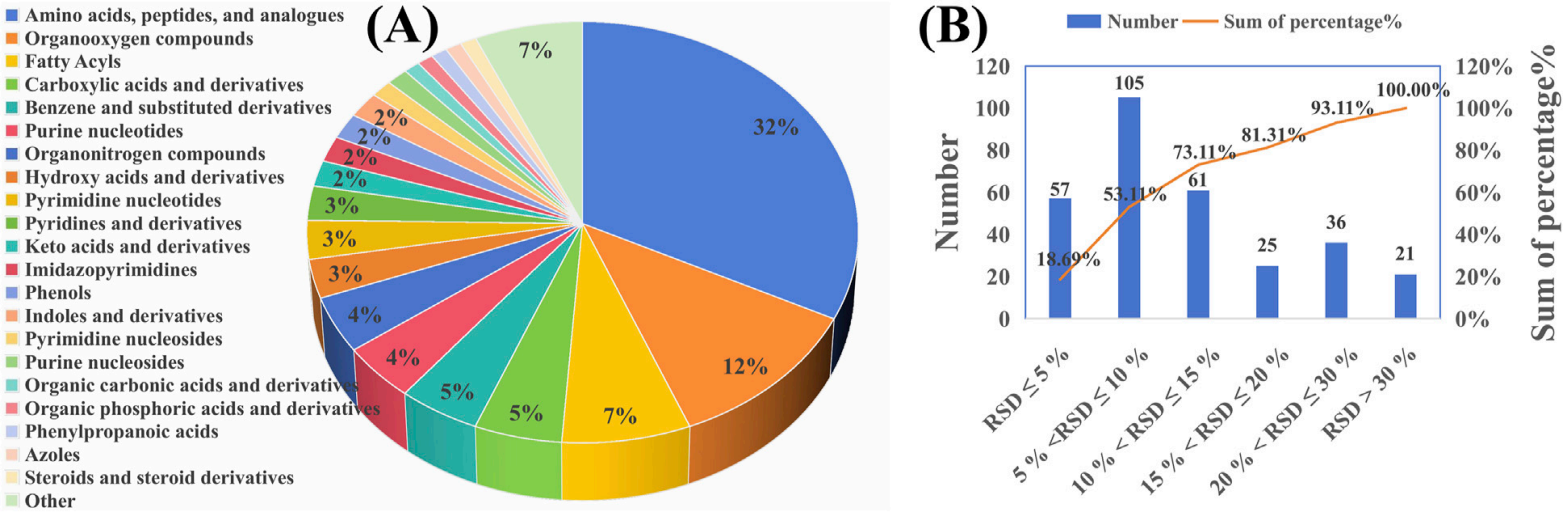
Metabolic profiling and quality control of the analyzed samples. **(A)** Pie chart representing the classification of all analyzed metabolites across different categories. **(B)** Bar graph showing the relative standard deviation (RSD) of peak areas for metabolites across six QC samples.

Alterations in amino acid, energy, and lipid metabolism are linked to ulcerative colitis (UC) (Wang et al., 2019). We developed an LC-MS/MS method using Dynamic MRM to monitor these changes. Figure 1A shows the distribution of compound classes: amino acids and peptides (32%), organic phosphoric acid derivatives (12%), carboxylic acids and derivatives (7%), fatty acyls (7%), benzene derivatives (6%), purine nucleotides (5%), pyrimidine nucleosides (5%), phenylpropanoic acids (5%), and steroids (4%). Other categories each contribute less than 5%. Quality control assessment, based on the relative standard deviation (RSD) of peak areas in six QC samples, showed that 81.31% of the metabolites had an RSD of less than 20% for peak area in the QC samples (n = 6) (Figure 1B), confirming the reliability and precision of the analytical methodology employed in this study.

### 3.2 Effects of berberine hydrochloride on DSS-induced colitis clinical symptoms of rat

**FIGURE 2:**
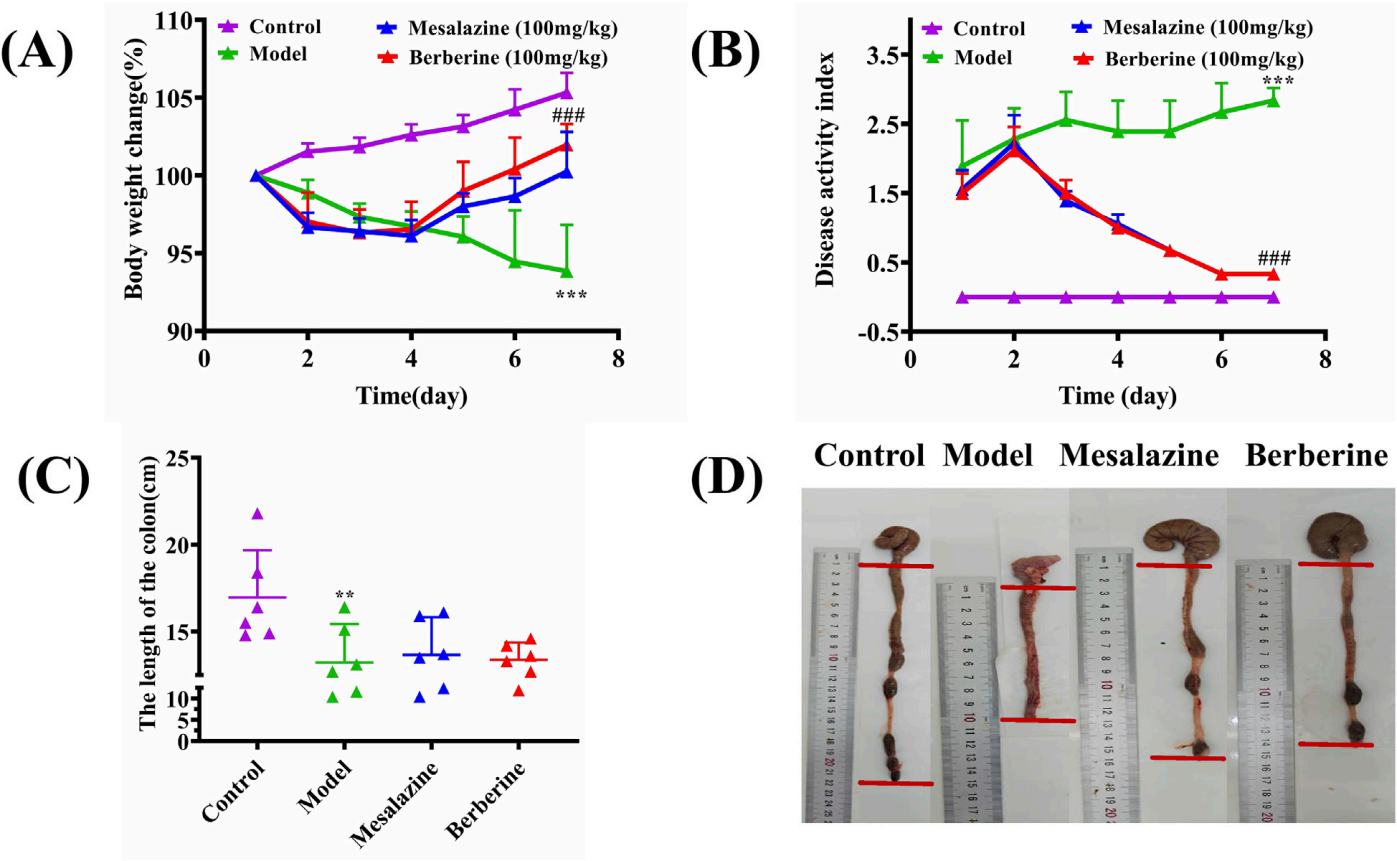
Berberine ameliorated the manifestations of DSS-induced ulcerative colitis. **(A)** Body weight change, **(B)** Disease activity index, **(C)** Colon length, **(D)** Representative appearance of the colon and rectum in each group. Data are expressed as mean ± SD (n = 6/group). The differences (in A, B, C) were analyzed using oneway ANOVA followed by Tukey’s *post hoc* tests (**p < 0.01, ***p < 0.001, between Model and Control group; ###*q* < 0.001, between Berberine and Model group).

Throughout the 7-day treatment period, rats in the Control group exhibited consistent weight gain, whereas those in the Model group experienced a notable weight decline. Both the Mesalazine and Berberine groups initially faced weight loss during the first 2 days of treatment; however, this trend decelerated over the subsequent 2 days and was succeeded by a steady increase during the final 3 days. These observations suggest that both berberine and mesalazine have the capacity to mitigate or potentially reverse the weight loss associated with ulcerative colitis (Figure 2A). The Disease Activity Index (DAI) was employed to evaluate the impact of UC on the rats. As depicted in Figure 2B, the DAI in the Model group showed a progressive increase relative to the Control group. Conversely, the DAI scores for both the Mesalazine and Berberine groups began to diminish by the second day of treatment, indicating that both interventions effectively alleviated the clinical symptoms associated with UC.

As illustrated in Figure 2C, the colon lengths of the Model group were markedly shorter compared to those of the Control, Berberine, and Mesalazine groups (Control group: 16.97 ± 2.48 cm; Model group: 13.22 ± 2.02 cm; Berberine group: 13.38 ± 0.90 cm; Mesalazine group: 13.67 ± 1.97 cm). Beyond the reduction in length, the colons in the Model group presented with evident signs of redness, swelling, and ulceration, contrasting sharply with those in the other groups (Figure 2D).

**FIGURE 3:**
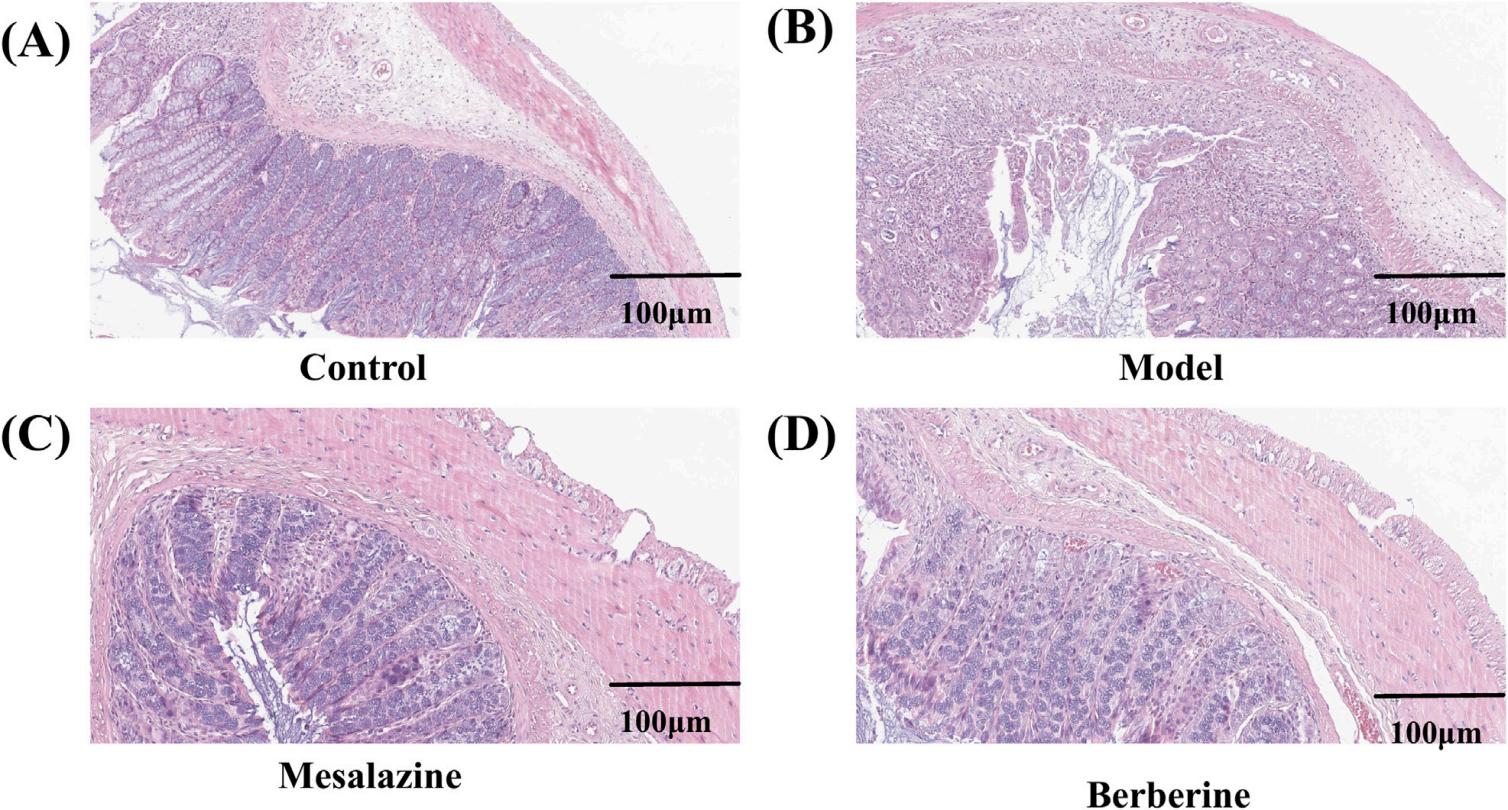
Representative pathological sections of colon tissues from rats in the Control **(A)**, Model **(B)**, Mesalazine **(C)**, and Berberine **(D)** groups. Scale bar, 100 µm.

Histological analysis was conducted to evaluate the extent of damage to the colon tissues. As shown in Figure 3A, hematoxylin and eosin (HE) staining of the Control group revealed no evidence of damage to the colonic mucosal epithelium. In stark contrast, the Model group exhibited extensive inflammatory cell infiltration within the submucosa, along with signs of crypt deformities (Figure 3B). However, the Mesalazine (Figure 3C) and Berberine (Figure 3D) groups demonstrated a marked reduction in congestion and edema compared to the Model group. Moreover, while the Model group displayed prominent inflammatory cell infiltrates and crypt abnormalities, the colons in both the Control and Berberine groups exhibited relatively intact crypt structures and epithelial integrity. These findings indicate that berberine can effectively attenuate the inflammation and colonic damage associated with ulcerative colitis.

### 3.3 Metabolite profiling analysis after berberine treatment

**FIGURE 4:**
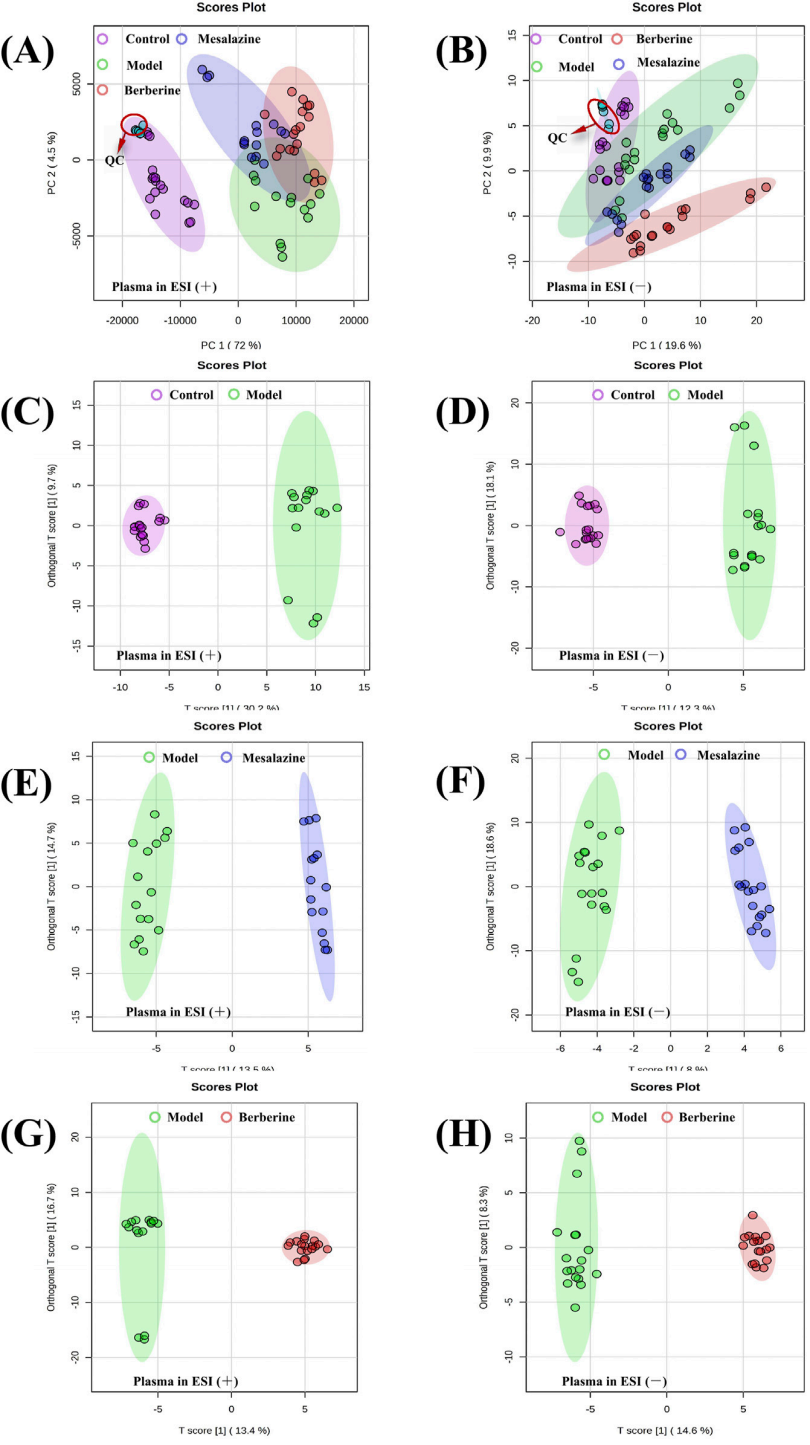
PCA and OPLS-DA score plots of plasma metabolic profiling between Control, Model, Mesalazine and Berberine group. PCA score plots of plasma detected in positive ESI mode **(A)** and negative ESI mode **(B)**. OPLS-DA score plots of plasma between Control and Model group **(C, D)**, Model and Mesalazine group **(E, F)**, Model and Berberine group **(G, H)**.

As depicted in Figure 4, principal component analysis (PCA) score plots reveal a tendency for the plasma metabolite profiles of the four groups to segregate. The tight clustering of QC samples in both positive (Figure 4A) and negative (Figure 4B) electrospray ionization (ESI) modes indicates excellent instrument stability and reproducibility, ensuring that the observed differences in metabolite profiles are attributed to biological variations rather than technical artifacts. Orthogonal projections to latent structures-discriminant analysis (OPLS-DA) models effectively distinguished between the Control and Model groups (Figures 4C, D), the Model and Mesalazine groups (Figures 4E, F), and the Model and Berberine groups (Figures 4G, H). Validation parameters of the OPLS-DA models, including Q^2^ and *R* ^2^ values, are provided in Supplementary Figure S1S, indicating a low risk of overfitting and high model reliability.

### 3.4 Altered metabolites related to the treatment group

**FIGURE 5:**
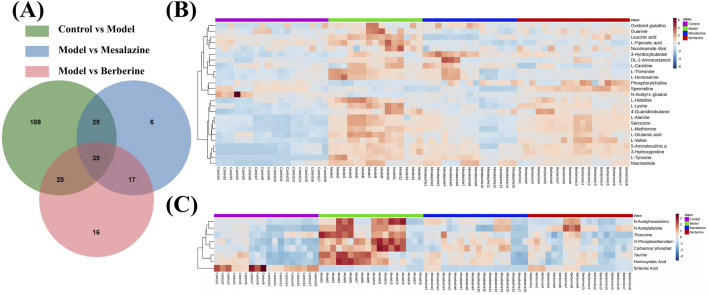
Venn diagrams **(A)** and heat maps **(B, C)** of the results of plasma metabolomics with LC-MS/MS. Heat maps reflecting the altered metabolites. Each cell in the heat map represents the fold change between the two groups: red colour represents an expression level above the mean and, blue colour represents an expression lower than the mean.

To elucidate the progression of UC and identify endogenous metabolites that exhibit significant positive changes following treatment. The preprocessing of the raw data was performed as described in Section 2.5. The VIP values, *p*-values, and fold change (FC) values were calculated using the statistics analysis functions in MetaboAnalyst 6.0. Three *p*-values was calculated: *p1* for the comparison between the Control group and the Model group (C/M), *p2* for the comparison between the Model group and the Mesalazine group (M/E), and *p3* for the comparison between the Model group and the Berberine group (M/B). ‘Mesalazine-Positive Impact Compounds’ were defined as those with both *p1* and *p2* were less than 0.05, with consistent trends in C/M and M/E comparisons. This indicated that the concentrations of these metabolites changed significantly in UC rats, suggesting that mesalazine tablets effectively modulate their levels to exert a protective effect. Similarly, ‘Berberine-Positive Impact Compounds’ were identified by both *p1* and *p3* were less than 0.05, with consistent trends between C/M and M/B comparisons. This suggested that berberine tablets can also directly regulate the levels of these metabolites to provide protection (Figure 5A). To ensure that no beneficial compounds from the treatment groups were overlooked, we screened for compounds under the criteria of VIP >1, *p* < 0.05, and fold change (FC) > 1.5 or FC < 2/3. The details of these compounds, which can be adjusted to exert protective roles, are summarized in Table 1.

**TABLE 1:** Statistical analysis results of the main metabolites changed in plasma (n = 6).

No	Name	Class	HMDB	Rt^a^ (min)	Control vs. model					Mesalazine vs. model					Berberine vs. model				
					VIP	FC^b^	log_2_ (FC)	*p*-value	Trend	VIP	FC	log_2_ (FC)	*p*-value	Trend	VIP	FC	log_2_ (FC)	*p*-value	Trend
1	*L*-Methionine	Amino acids, peptides, and analogues	HMDB0000696	1.79	1.72	0.54	−0.9	<0.001	↓^**^	2.07	0.68	−0.55	<0.001	↓^**^	0.72	0.96	−0.06	-	↓
2	3-Hydroxyproline		NA	17.76	1.7	0.04	−4.58	<0.001	↓^**^	1.85	0.06	−4.11	<0.001	↓^**^	1.81	0.04	−4.53	<0.001	↓^**^
3	5-Aminolevulinic acid		HMDB0001149	2.56	1.71	0.15	−2.74	<0.001	↓^**^	1.66	0.62	−0.68	<0.001	↓^**^	0.23	0.86	−0.22	-	↓
4	Sarcosine		HMDB0000271	1.34	1.63	0.56	−0.84	<0.001	↓^**^	1.88	0.73	−0.46	<0.001	↓^**^	0.74	0.94	−0.08	-	↓
5	*L*-Valine		HMDB0000883	1.54	1.7	0.51	−0.96	<0.001	↓^**^	1.79	0.72	−0.47	<0.001	↓^**^	1.09	1.1	0.14	0.013	↑^*^
6	*L*-Alanine		HMDB0000161	1.33	1.53	0.12	−3.1	<0.001	↓^**^	1.69	0.73	−0.45	<0.001	↓^**^	1.12	1.19	0.26	0.0099	↑^**^
7	4-Guanidinobutanoic acid		HMDB0003464	1.45	1.42	0.51	−0.97	<0.001	↓^**^	1.3	0.77	−0.37	0.005	↓^**^	0.32	0.93	−0.11	-	↓
8	*L*-Tyrosine		HMDB0000158	4.22	1.67	0	−8.14	<0.001	↓^**^	1.02	0.78	−0.36	-	↓	1.43	0.69	−0.54	0.002	↓^**^
9	*L*-Homoserine		HMDB0000719	1.12	1.47	0.66	−0.6	<0.001	↓^**^	1.2	0.8	−0.32	-	↓	2.12	0.71	−0.5	<0.001	↓^**^
10	*DL*-2-Aminooctanoic acid		HMDB0000991	1.23	1.22	0.61	−0.72	<0.001	↓^**^	0.51	0.92	−0.11	-	↓	1.24	0.75	−0.42	0.009	↓^**^
11	N-Acetyl-L-glutamic acid		HMDB0001138	NA	0.87	2.78	1.48	0.005	↑^**^	0.55	1.17	0.22	-	↑	1.97	1.76	0.81	<0.001	↑^**^
12	Oxidized glutathione		HMDB0003337	NA	1.15	0.07	−3.86	<0.001	↓^**^	0.36	0.8	−0.33	-	↓	1.33	0.51	−0.96	0.007	↓^**^
13	Homocysteic Acid		HMDB0002205	1.07	1.95	0.65	−0.61	<0.001	↓^**^	1.86	0.75	−0.41	<0.001	↓^**^	1.68	0.7	−0.52	<0.001	↓^**^
14	Threonine		HMDB0000167	1.07	1.82	0.7	−0.51	<0.001	↓^**^	2.03	0.73	−0.46	<0.001	↓^**^	1.71	0.64	−0.65	<0.001	↓^**^
15	*L*-Glutamic acid		HMDB0000148	1.14	1.57	0.63	−0.67	<0.001	↓^**^	2.18	0.71	−0.48	<0.001	↓^**^	1.97	1.76	0.81	<0.001	↑^**^
16	*L*-Threonine		HMDB0000167	1.13	1.52	0.66	−0.59	<0.001	↓^**^	1.26	0.79	−0.34	<0.001	↓^**^	2.18	0.73	−0.45	<0.001	↓^**^
17	*L*-Lysine		HMDB0000182	0.94	1.59	0.63	−0.67	<0.001	↓^**^	2.15	0.65	−0.63	<0.001	↓^**^	1.18	0.86	−0.21	<0.001	↓^**^
18	*L*-Histidine		HMDB0000177	1.12	1.4	0.78	−0.37	<0.001	↓^**^	1.99	0.79	−0.33	<0.001	↓^**^	1.33	0.9	−0.16	0.01	↓^*^
19	*L*-Pipecolic acid		HMDB0000716	NA	1.06	0.73	−0.45	<0.001	↓^**^	2.28	0.46	−1.11	<0.001	↓^**^	2.14	0.51	−0.98	<0.001	↓^**^
20	3-Hyrdoxybutarate	NA	NA	1.1	0.65	0.88	−0.18	<0.001	↓^**^	1.4	1.29	0.37	0.005	↑^**^	0.41	0.96	−0.06	-	↓
21	Guanine	Imidazopyrimidines	HMDB0000132	NA	0.84	1.75	0.8	<0.001	↑^**^	1.53	0.56	−0.84	0.002	↓^**^	0.92	0.72	−0.47	-	↓
22	Nicotinamide ribotide	Pyridine nucleotides	HMDB0000229	NA	0.66	0.67	−0.58	0.022	↓^*^	1.25	0.61	−0.72	0.012	↓^*^	0.49	0.83	−0.26	-	↓
23	Niacinamide	Pyridines and derivatives	HMDB0001406	1.5	1.52	0.59	−0.76	<0.001	↓^**^	0.42	0.95	−0.08	-	↓	1.54	0.83	−0.27	0.001	↓^**^
24	*L*-Carnitine	Organonitrogen compounds	HMDB0000062	1.15	0.81	0.85	−0.23	0.01015	↓^*^	0.49	1.08	0.11	-	↑	1.64	0.74	−0.43	<0.001	↓^**^
25	Phosphorylcholine		HMDB0001565	NA	1.31	1.91	0.94	<0.001	↑^**^	1.69	2.15	1.1	<0.001	↑^**^	1.82	2.79	1.48	<0.001	↑^**^
26	Spermidine		HMDB0001257	0.88	1.01	1.29	0.37	0.002	↑^**^	1.63	0.73	−0.46	<0.001	↓^**^	2.25	1.61	0.69	<0.001	↑^**^
27	N-Acetylalanine	Carboxylic acids and derivatives	HMDB0000766	1.03	2.15	0.57	−0.81	<0.001	↓^**^	2.02	0.65	−0.62	<0.001	↓^**^	0.63	0.87	−0.19	-	↓
28	Carbamoyl phosphate	Organic phosphoric acids and derivatives	HMDB0001096	1.07	2.11	0.58	−0.79	<0.001	↓^**^	1.71	0.76	−0.4	<0.001	↓^**^	1.65	0.66	−0.59	<0.001	↓^**^
29	*O*-Phosphoethanolamine		HMDB0000224	1.07	2.06	0.56	−0.83	<0.001	↓^**^	1.84	0.74	−0.43	0.002	↓^**^	1.59	0.66	−0.59	<0.001	↓^**^
30	Taurine		HMDB0000251	1.08	1.89	0.66	−0.61	<0.001	↓^**^	1.73	0.76	−0.4	0.001	↓^**^	1.47	0.73	−0.46	<0.001	↓^**^
31	Leucinic acid	Fatty Acyls	HMDB0000665	NA	1.4	0.61	−0.71	<0.001	↓^**^	2.31	0.34	−1.54	<0.001	↓^**^	1.92	0.6	−0.73	<0.001	↓^**^
32	N-Acetylneuraminic acid	Organooxygen compounds	HMDB0000230	NA	1.63	0.63	−0.68	<0.001	↓^**^	1.37	0.75	−0.41	-	↓	1.26	0.66	−0.61	0.003	↓^**^
33	Shikimic Acid		HMDB0003070	2.60	2.25	6.93	2.79	<0.001	↑^**^	0.17	0.96	−0.05	-	↓	1.09	2.31	1.21	0.01	↑^*^

^a^ Rt: The retention time of the sample.

^b^ FC: fold change, as determined by average relative quantitation obtained from group 1/Model, log_2_ (FC) more than 0 indicates an increase (↑) in group 1, log_2_ (FC) less than 0 indicates a decrease (↓) in group1. Control vs. Model, group 1 = Control; Mesalazine vs. Model, group 1 = Mesalazine; Berberine vs. Model, group 1 = Berberine. The “-” indicates that the corresponding metabolite did not pass through the screening process. *, *p* < 0.05; **, *p* < 0.01.

After excluding non-endogenous compounds, a total of 33 altered metabolites were identified in the plasma, primarily categorized as amino acids, pyrimidines, organic phosphoric acids, fatty acyls, and organonitrogen compounds. Heat maps were employed to illustrate the trends in metabolite changes (Figures 5B, C). Disruptions in amino acid metabolism, purine metabolism, vitamin metabolism, and lipid metabolism in both urine and feces are closely associated with the onset of UC (Liao et al., 2019). Notably, while amino acid compounds in plasma exhibited the greatest variability, changes in the concentrations of pyrimidines, organic phosphoric acids, fatty acyl groups, and organonitrogen compounds have been less frequently reported in prior studies. Despite challenges in obtaining standards, a standard reference solution was used to compare the chromatographic peaks of the 16 compounds (Supplementary Figures S2S, S3S).

**FIGURE 6:**
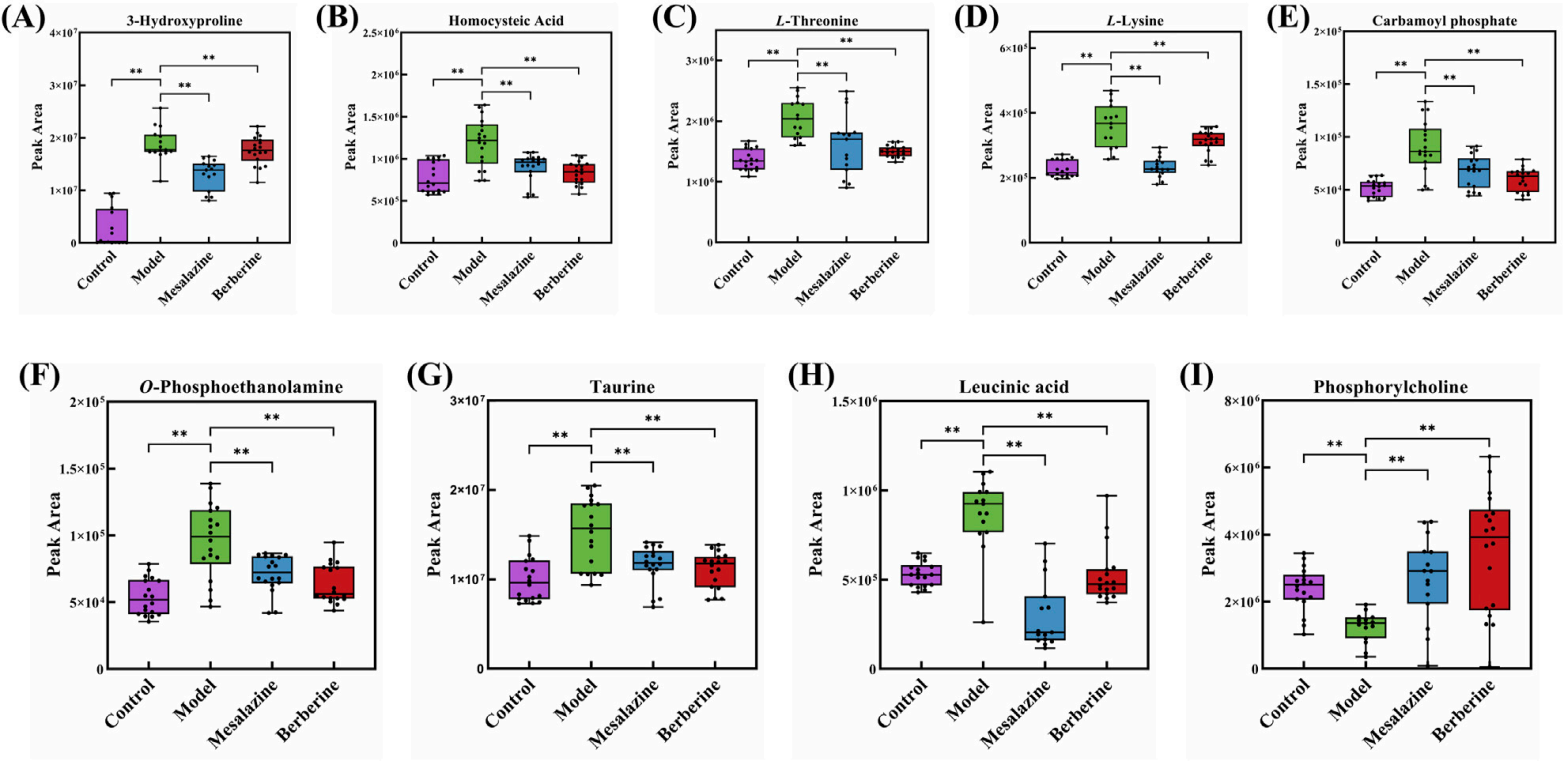
Peak area of 3-Hydroxyproline **(A)**, Homocysteic Acid **(B)**, *L*-Threonine **(C)**, *L*-Lysine **(D)**, Carbamoyl phosphate **(E)**, *O*-Phosphoethanolamine **(F)**, Taurine **(G)**, Leucinic acid **(H)** and Phosphorylcholine **(I)** in Control group, Model group, Mesalazine group and Berberine group. (**p < 0.001).

Endoscopy combined with biopsy remains the gold standard for diagnosing and managing UC. However, imaging techniques have also been developed for monitoring the condition. These methods can be costly and invasive, often causing patient discomfort (Liu et al., 2022). Sensitive and specific biomarkers are therefore essential for the diagnosis and treatment of UC. Notably, metabolites such as 3-hydroxyproline, homocysteic Acid, *L*-threonine, *L*-lysine, carbamoyl phosphate, *O*-phosphoethanolamine, taurine, leucinic acid, and phosphorylcholine exhibited significant differences between the Treatment group and the Model group (*p* < 0.001), with a trend indicating a return toward Control group levels (Figure 6). These findings suggest that these compounds may serve as potential plasma biomarkers for UC.

### 3.5 Metabolic pathway analysis

Based on the plasma metabolomics results (Table 1) and KEGG pathway analysis (Table 2), both berberine and mesalazine exert therapeutic effects against UC via several metabolic pathways, including arginine biosynthesis, glutathione metabolism, alanine, aspartate, and glutamate metabolism, nicotinate and nicotinamide metabolism, glycine, serine, and threonine metabolism, taurine and hypotaurine metabolism, pyrimidine metabolism, and glyoxylate and dicarboxylate metabolism. However, since specific metabolites such as *L*-carnitine, spermidine, and shikimic acid were only restored in the berberine treatment group, this suggested that pathways such as the citric acid cycle (TCA cycle) may be uniquely involved in berberine’s anti-UC mechanism.

**TABLE 2:** Statistical analysis results of metabolic pathway.

NO.	Name	Model vs. mesalazine					Model vs. berberine				
		Total	Hits	p	-log_10_(p)	Impact	Total	Hits	p	-log_10_(p)	Impact
1	Arginine biosynthesis	14	5	<0.01	4.46	0.32	14	5	<0.01	4.17	0.32
2	Glutathione metabolism	28	6	<0.01	3.88	0.15	28	6	<0.01	3.54	0.15
3	Alanine, aspartate and glutamate metabolism	28	4	0.01	2.04	0.25	28	4	0.01	1.84	0.25
4	Nicotinate and nicotinamide metabolism	15	3	0.01	2.03	0.23	15	3	0.01	1.87	0.23
5	Glycine, serine and threonine metabolism	33	4	0.02	1.79	0.35	33	5	0.00	2.31	0.35
6	Taurine and hypotaurine metabolism	8	2	0.02	1.64	0.43	8	2	0.03	1.53	0.43
7	Pyrimidine metabolism	39	4	0.03	1.54	0.13	39	5	0.01	2.00	0.16
8	Glyoxylate and dicarboxylate metabolism	32	3	0.07	1.15	0.11	32	4	0.02	1.64	0.13
9	Histidine metabolism	16	2	0.08	1.08	0.22	-	-	-	-	-
10	Citrate cycle (TCA cycle)	-	-	-	-	-	20	3	0.03	1.52	0.15

## 4 Discussion

In this study, a comprehensive method for monitoring small molecules in rat plasma was developed using LC-MS/MS-based Dynamic MRM. This approach offers broad coverage of compounds, low detection limits, and high precision, making it suitable for detailed metabolic profiling. The method was successfully applied to monitor changes in metabolites following treatment with berberine and mesalazine, demonstrating its effectiveness in detecting subtle metabolic alterations induced by these treatments. Compared to UHPLC-HRMS-based broad-target metabolomics, this method simplifies data processing, which enhances the efficiency and reproducibility of the analysis. Additionally, it outperforms targeted metabolomics in terms of the number of compounds detected, thereby providing a more comprehensive overview of the metabolome. The broad coverage and high sensitivity of this method make it a valuable tool for identifying potential biomarkers and elucidating the mechanisms of action of therapeutic interventions in various disease models. Furthermore, the versatility and robustness of this approach hold significant promise for future applications in clinical diagnostics, drug development, and personalized medicine, where precise and comprehensive metabolic profiling is essential.

Mesalazine, a well-established drug for ulcerative colitis (UC), has a long history of reducing inflammation and promoting mucosal healing, as documented in numerous clinical trials (Paridaens et al., 2021). Berberine, a natural alkaloid, has emerged as a promising alternative for UC due to its anti-inflammatory and immunomodulatory properties (Zhu et al., 2022). In this study, both mesalazine and berberine effectively modulated specific metabolic pathways, but with distinct patterns. Berberine showed a trend toward normalizing *L*-carnitine, spermidine, and shikimic acid levels, while mesalazine exhibited a trend toward normalizing *L*-valine, *L*-alanine, and *L*-glutamic acid levels. These differences suggest that the two compounds influence distinct metabolic pathways. Mesalazine specifically affects histidine metabolism, which is crucial for maintaining intestinal barrier function and regulating immune responses. Altered histidine metabolism has been linked to increased intestinal permeability and inflammation, key features of UC (Jagt et al., 2022). Berberine impacts the citrate cycle (TCA cycle), a central pathway in energy production and intermediate synthesis. Dysregulation of the TCA cycle can lead to impaired energy metabolism and oxidative stress, contributing to UC pathogenesis (Connors et al., 2018). These findings highlight the unique mechanisms of action of mesalazine and berberine, providing insights into their potential for targeted UC therapy and guiding the development of more personalized treatment strategies.

## 5 Conclusion

In conclusion, our study sought to elucidate the relationship between metabolism, UC, and the therapeutic effects of berberine. The results demonstrated that berberine effectively mitigated clinical symptoms associated with UC and exhibited a protective effect on the colonic tissue of affected rats. Metabolomics analysis revealed that berberine modulated metabolic disturbances involving amino acids, pyrimidines, organic phosphoric acids, fatty acyls, and organonitrogen compounds in the plasma of UC rats. Notably, compounds such as 3-hydroxyproline, homocysteic acid, *L*-threonine, *L*-lysine, carbamoyl phosphate, *O*-phosphoethanolamine, taurine, leucine, and phosphorylcholine exhibited significant differences between the Treatment and Model groups, trending back toward levels observed in the Control group (*p* ˂ 0.001). These metabolites not only reflect the disease state but also the efficacy of the treatment. This dual role enhances their potential value as plasma biomarkers for UC. Beyond its influence on amino acid metabolism, berberine also regulated the body’s antioxidant systems, vitamin pathways, lipid metabolism, oxidative stress responses, and other signaling pathways, thereby addressing the metabolic disturbances associated with UC. This study provided a theoretical foundation for understanding the therapeutic mechanisms of berberine in treating UC.

## Data Availability

The original contributions presented in the study are included in the article/Supplementary Material, further inquiries can be directed to the corresponding author.

## Supplementary material

SUPPLEMENTARY FIGURE S1 Comparative analysis of model performance across different treatments under both positive (ESI+) and negative (ESI-) ionization modes. Panels A through F depict R^2^ and Q^2^ values for various experimental conditions, including Control and Model group **(A, D)**, Model and Mesalazine group **(B, E)**, Model and Berberine group **(C, F)** comparisons. Green circles represent *R* ^2^, blue squares represent Q^2^, with dashed lines indicating expected values from permutation tests.

SUPPLEMENTARY FIGURE S2 Chromatograms of *L*-Methionine **(A)**, Sarcosine **(B)**, *L*-Valine **(C)**, *L*-Alanine **(D)**, *DL*-2-Aminooctanoic acid **(E)**, *L*-Glutamic acid **(F)**, *L*-Threonine **(G)**, *L*-Lysine **(H)**, *L*-Histidine **(I)** in plasma samples and reference solution.

SUPPLEMENTARY FIGURE S3 Chromatograms of *L*-Pipecolic acid **(A)**, Guanine **(B)**, Niacinamide **(C)**, *O*-Phosphoethanolamine **(D)**, Taurine **(E)**, *L*-Carnitine **(F)**, Spermidine **(G)** plasma samples and reference solution.

## References

[B1] Aloi M. Cucchiara S. (2009). Extradigestive manifestations of IBD in pediatrics. Eur. Rev. Med. Pharmacol. Sci. 13 (Suppl. 1), 23–32. 19530508

[B2] Bujak R. Struck-Lewicka W. Markuszewski M. J. Kaliszan R. (2015). Metabolomics for laboratory diagnostics. J. Pharm. Biomed. Anal. 113, 108–120. 10.1016/j.jpba.2014.12.017 25577715

[B3] Chen H. Zhang J. Zhou H. Zhu Y. Liang Y. Zhu P. (2022). UHPLC-HRMS-based serum lipisdomics reveals novel biomarkers to assist in the discrimination between colorectal adenoma and cancer. Front. Oncol. 12, 934145. 10.3389/fonc.2022.934145 35965551 PMC9366052

[B4] Chen H. Zhou H. Liang Y. Huang Z. Yang S. Wang X. (2023). UHPLC-HRMS-based serum untargeted lipidomics: phosphatidylcholines and sphingomyelins are the main disturbed lipid markers to distinguish colorectal advanced adenoma from cancer. J. Pharm. Biomed. Anal. 234, 115582. 10.1016/j.jpba.2023.115582 37473505

[B5] Chen L. Zhong F. Zhu J. (2020). Bridging targeted and untargeted mass spectrometry-based metabolomics via hybrid approaches. Metabolites 10, 348. 10.3390/metabo10090348 32867165 PMC7570162

[B6] Chen M. X. Wang S. Y. Kuo C. H. Tsai I. L. (2019). Metabolome analysis for investigating host-gut microbiota interactions. J. Formos. Med. Assoc. 118 (Suppl. 1), S10–s22. 10.1016/j.jfma.2018.09.007 30269936

[B7] Chen S. Kong H. Lu X. Li Y. Yin P. Zeng Z. (2013). Pseudotargeted metabolomics method and its application in serum biomarker discovery for hepatocellular carcinoma based on ultra high-performance liquid chromatography/triple quadrupole mass spectrometry. Anal. Chem. 85, 8326–8333. 10.1021/ac4016787 23889541

[B8] Connors J. Dawe N. Van Limbergen J. (2018). The role of succinate in the regulation of intestinal inflammation. Nutrients 11, 25. 10.3390/nu11010025 30583500 PMC6356305

[B9] Demirhan D. Kumar A. Zhu J. Poulsen P. C. Majewska N. I. Sebastian Y. (2022). Comparative systeomics to elucidate physiological differences between CHO and SP2/0 cell lines. Sci. Rep. 12, 3280. 10.1038/s41598-022-06886-1 35228567 PMC8885639

[B10] Deng C. Zhang H. Li Y. Cheng X. Liu Y. Huang S. (2024). Exosomes derived from mesenchymal stem cells containing berberine for ulcerative colitis therapy. J. Colloid Interface Sci. 671, 354–373. 10.1016/j.jcis.2024.05.162 38815372

[B11] De Oliveira Madeira J. L. Antoneli F. (2024). Homeostasis in networks with multiple inputs. J. Math. Biol. 89, 17. 10.1007/s00285-024-02117-5 38902549 PMC11190020

[B12] Emwas A. H. (2015). The strengths and weaknesses of NMR spectroscopy and mass spectrometry with particular focus on metabolomics research. Methods Mol. Biol. 1277, 161–193. 10.1007/978-1-4939-2377-9_13 25677154

[B13] Fabián O. Kamaradová K. (2022). Morphology of inflammatory bowel diseases (IBD). Cesk Patol. 58, 27–37. 35387455

[B14] Grasso D. Geminiani M. Galderisi S. Iacomelli G. Peruzzi L. Marzocchi B. (2022). Untargeted NMR metabolomics reveals alternative biomarkers and pathways in alkaptonuria. Int. J. Mol. Sci. 23, 15805. 10.3390/ijms232415805 36555443 PMC9779518

[B15] Grogan K. E. Perry G. H. (2020). Studying human and nonhuman primate evolutionary biology with powerful *in vitro* and *in vivo* functional genomics tools. Evol. Anthropol. 29, 143–158. 10.1002/evan.21825 32142200 PMC10574139

[B16] Huang M. Zhou T. (2022). Comprehensive pseudotargeted metabolomics analysis based on two-phase liquid extraction-UHPLC-MS/MS for the investigation of depressive rats. J. Sep. Sci. 45, 2977–2986. 10.1002/jssc.202200255 35648513

[B17] Jagt J. Z. Struys E. A. Ayada I. Bakkali A. Jansen E. E. W. Claesen J. (2022). Fecal amino acid analysis in newly diagnosed pediatric inflammatory bowel disease: a multicenter case-control study. Inflamm. Bowel Dis. 28, 755–763. 10.1093/ibd/izab256 34757415 PMC9074868

[B18] Liao Z. Zhang S. Liu W. Zou B. Lin L. Chen M. (2019). LC-MS-based metabolomics analysis of Berberine treatment in ulcerative colitis rats. J. Chromatogr. B Anal. Technol. Biomed. Life Sci. 1133, 121848. 10.1016/j.jchromb.2019.121848 31756623

[B19] Liu B. Du Z. Zhang W. Guo X. Lu Y. Jiang Y. (2024). A pseudo-targeted metabolomics for discovery of potential biomarkers of cardiac hypertrophy in rats. J. Chromatogr. B Anal. Technol. Biomed. Life Sci. 1240, 124133. 10.1016/j.jchromb.2024.124133 38733887

[B20] Liu D. Saikam V. Skrada K. A. Merlin D. Iyer S. S. (2022). Inflammatory bowel disease biomarkers. Med. Res. Rev. 42, 1856–1887. 10.1002/med.21893 35603998 PMC10321231

[B21] Luo P. Dai W. Yin P. Zeng Z. Kong H. Zhou L. (2015). Multiple reaction monitoring-ion pair finder: a systematic approach to transform nontargeted mode to pseudotargeted mode for metabolomics study based on liquid chromatography-mass spectrometry. Anal. Chem. 87, 5050–5055. 10.1021/acs.analchem.5b00615 25884293

[B22] Ma J. Li K. Shi S. Li J. Tang S. Liu L. (2022). The application of UHPLC-HRMS for quality control of traditional Chinese medicine. Front. Pharmacol. 13, 922488. 10.3389/fphar.2022.922488 35721122 PMC9201421

[B23] Moco S. (2022). Studying metabolism by NMR-based metabolomics. Front. Mol. Biosci. 9, 882487. 10.3389/fmolb.2022.882487 35573745 PMC9094115

[B24] Paridaens K. Fullarton J. R. Travis S. P. L. (2021). Efficacy and safety of oral Pentasa (prolonged-release mesalazine) in mild-to-moderate ulcerative colitis: a systematic review and meta-analysis. Curr. Med. Res. Opin. 37, 1891–1900. 10.1080/03007995.2021.1968813 34404286

[B25] Peyrin-Biroulet L. Panés J. Sandborn W. J. Vermeire S. Danese S. Feagan B. G. (2016). Defining disease severity in inflammatory bowel diseases: current and future directions. Clin. Gastroenterol. Hepatol. 14, 348–354.e17. 10.1016/j.cgh.2015.06.001 26071941

[B26] Schrimpe-Rutledge A. C. Codreanu S. G. Sherrod S. D. McLean J. A. (2016). Untargeted metabolomics strategies-challenges and emerging directions. J. Am. Soc. Mass Spectrom. 27, 1897–1905. 10.1007/s13361-016-1469-y 27624161 PMC5110944

[B27] Song D. Hao J. Fan D. (2020). Biological properties and clinical applications of berberine. Front. Med. 14, 564–582. 10.1007/s11684-019-0724-6 32335802

[B28] Tavassoly I. Goldfarb J. Iyengar R. (2018). Systems biology primer: the basic methods and approaches. Essays Biochem. 62, 487–500. 10.1042/ebc20180003 30287586

[B29] Temby M. Boye T. L. Hoang J. Nielsen O. H. Gubatan J. (2023). Kinase signaling in colitis-associated colon cancer and inflammatory bowel disease. Biomolecules 13, 1620. 10.3390/biom13111620 38002302 PMC10669043

[B30] Wang C. R. Tsai H. W. (2023). Seronegative spondyloarthropathy-associated inflammatory bowel disease. World J. Gastroenterol. 29, 450–468. 10.3748/wjg.v29.i3.450 36688014 PMC9850936

[B31] Wang X. Guan X. Tong Y. Liang Y. Huang Z. Wen M. (2024). UHPLC-HRMS-based multiomics to explore the potential mechanisms and biomarkers for colorectal cancer. BMC Cancer 24, 644. 10.1186/s12885-024-12321-7 38802800 PMC11129395

[B32] Wang Y. Tang H. Yang X. Wang L. (2019). Metabolic profiling reveals altered amino acid, energy, and lipid metabolism in ulcerative colitis. J. Proteome Res. 18, 235–245. 10.1007/s11306-017-1311-y

[B33] Wei Y. Jasbi P. Shi X. Turner C. Hrovat J. Liu L. (2021). Early breast cancer detection using untargeted and targeted metabolomics. J. Proteome Res. 20, 3124–3133. 10.1021/acs.jproteome.1c00019 34033488

[B34] Yuan M. Breitkopf S. B. Yang X. Asara J. M. (2012). A positive/negative ion-switching, targeted mass spectrometry-based metabolomics platform for bodily fluids, cells, and fresh and fixed tissue. Nat. Protoc. 7, 872–881. 10.1038/nprot.2012.024 22498707 PMC3685491

[B35] Zheng F. Zhao X. Zeng Z. Wang L. Lv W. Wang Q. (2020). Development of a plasma pseudotargeted metabolomics method based on ultra-high-performance liquid chromatography-mass spectrometry. Nat. Protoc. 15, 2519–2537. 10.1038/s41596-020-0341-5 32581297

[B36] Zhu C. Li K. Peng X. X. Yao T. J. Wang Z. Y. Hu P. (2022). Berberine a traditional Chinese drug repurposing: its actions in inflammation-associated ulcerative colitis and cancer therapy. Front. Immunol. 13, 1083788. 10.3389/fimmu.2022.1083788 36561763 PMC9763584

