# Supplementary material for: Comprehensive plasma metabolomics analysis of berberine treatment in ulcerative colitis rats by LC-MS/MS
Source: Front Chem. 2024 Dec 11;12:1518110. doi: 10.3389/fchem.2024.1518110 (PMC11668600; doi:10.3389/fchem.2024.1518110)
Supplement: Supplementary file 1 [file DataSheet1.docx]

Supplementary Material

**Table S1** Scoring System for Disease Activity Index (DAI)

| **Parameter** | **Score** | **Description** |
| --- | --- | --- |
| Body Weight Loss | 0 | No weight loss |
|  | 1 | 1-5% weight loss |
|  | 2 | 5-10% weight loss |
|  | 3 | 10-15% weight loss |
|  | 4 | More than 15% weight loss |
| Stool Consistency | 0 | Normal |
|  | 2 | Loose stools |
|  | 4 | Diarrhea |
| Occult Blood | 0 | No bleeding |
|  | 2 | Positive for occult blood |
|  | 4 | Visible bleeding |


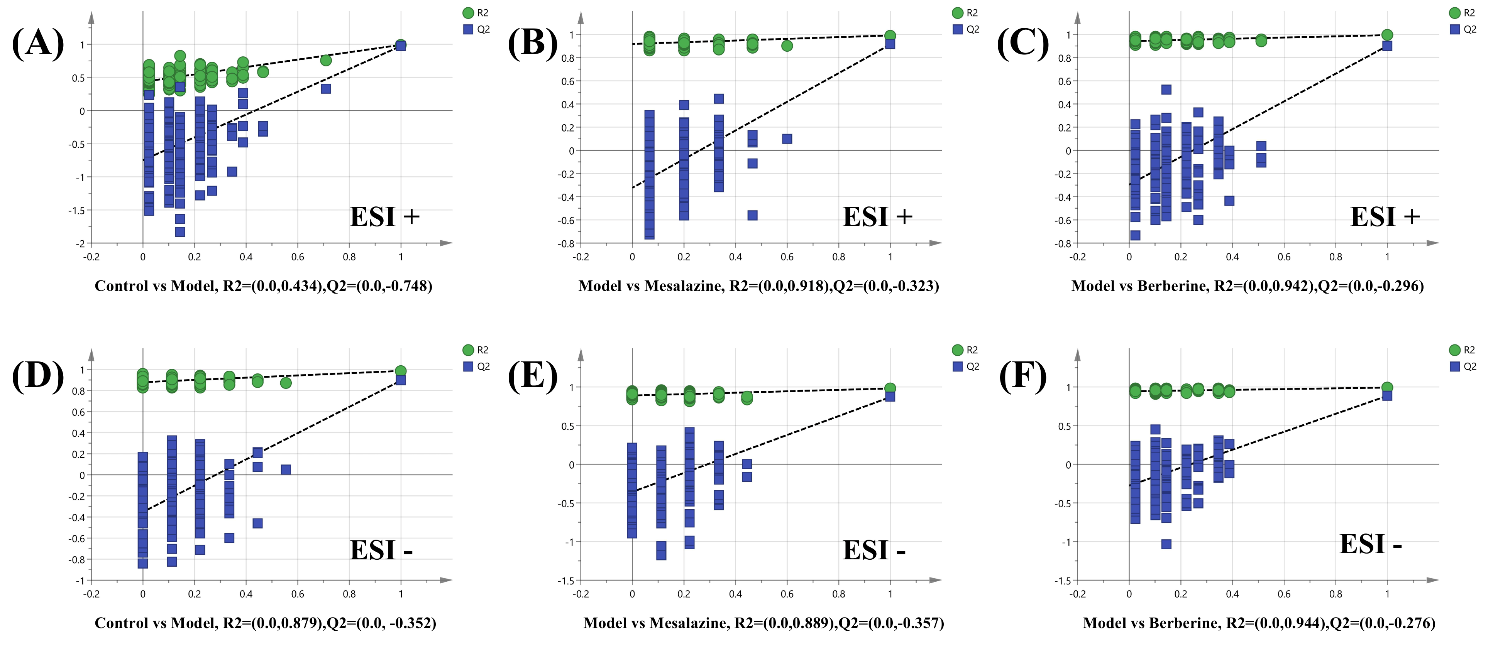


**Fig. 1s** Comparative analysis of model performance across different treatments under both positive (ESI+) and negative (ESI-) ionization modes. Panels A through F depict R² and Q² values for various experimental conditions, including control vs. model (A, D), model vs. mesalazine (B, E), and model vs. berberine (C, F) comparisons. Green circles represent R², blue squares represent Q², with dashed lines indicating expected values from permutation tests.


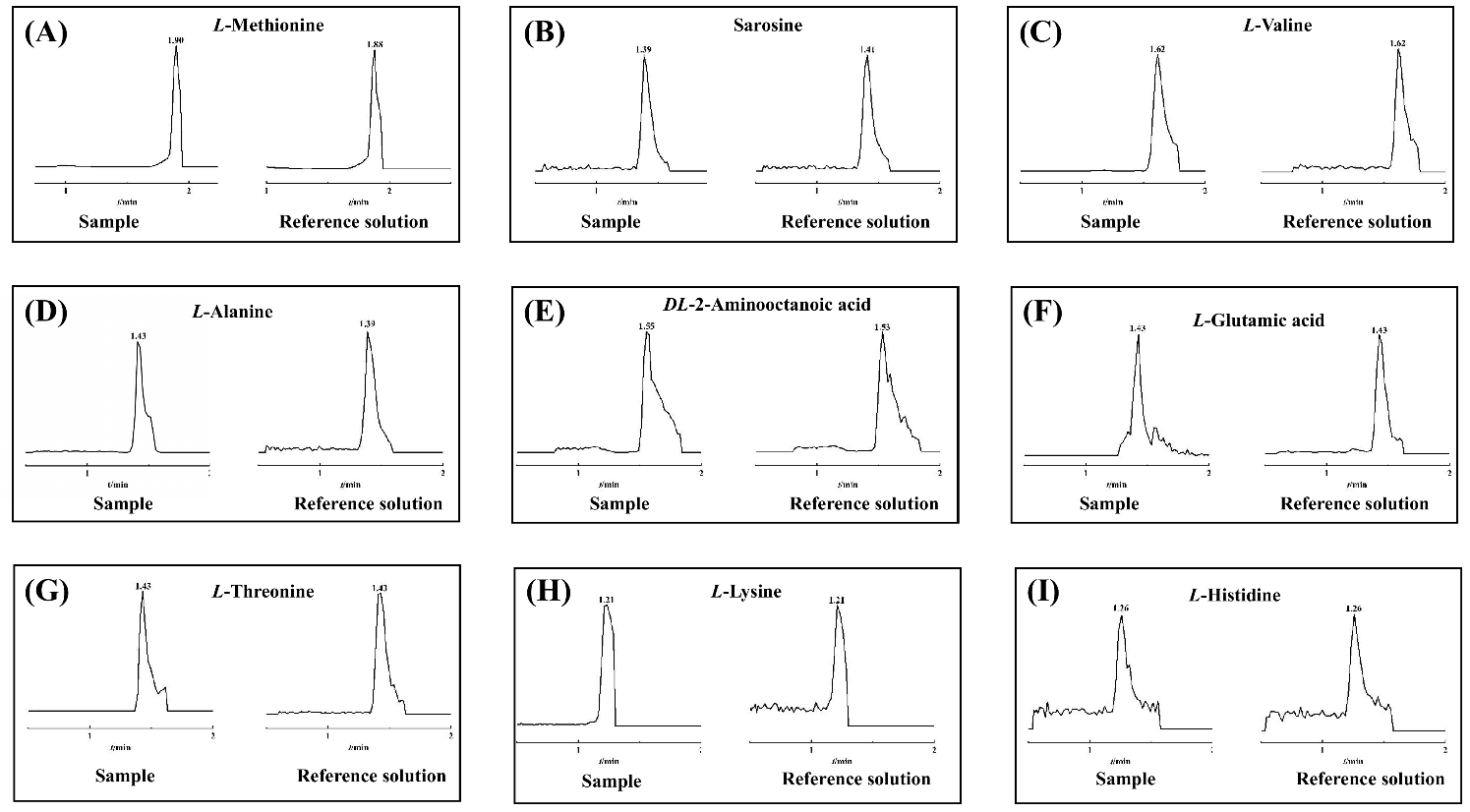


**Fig. 2S** Chromatograms of *L*-Methionine (A), Sarcosine (B), *L*-Valine (C), *L*-Alanine (D), *DL*-2-Aminooctanoic acid (E), *L*-Glutamic acid (F), *L*-Threonine (G), *L*-Lysine (H), *L*-Histidine (I) in plasma samples and reference solution.


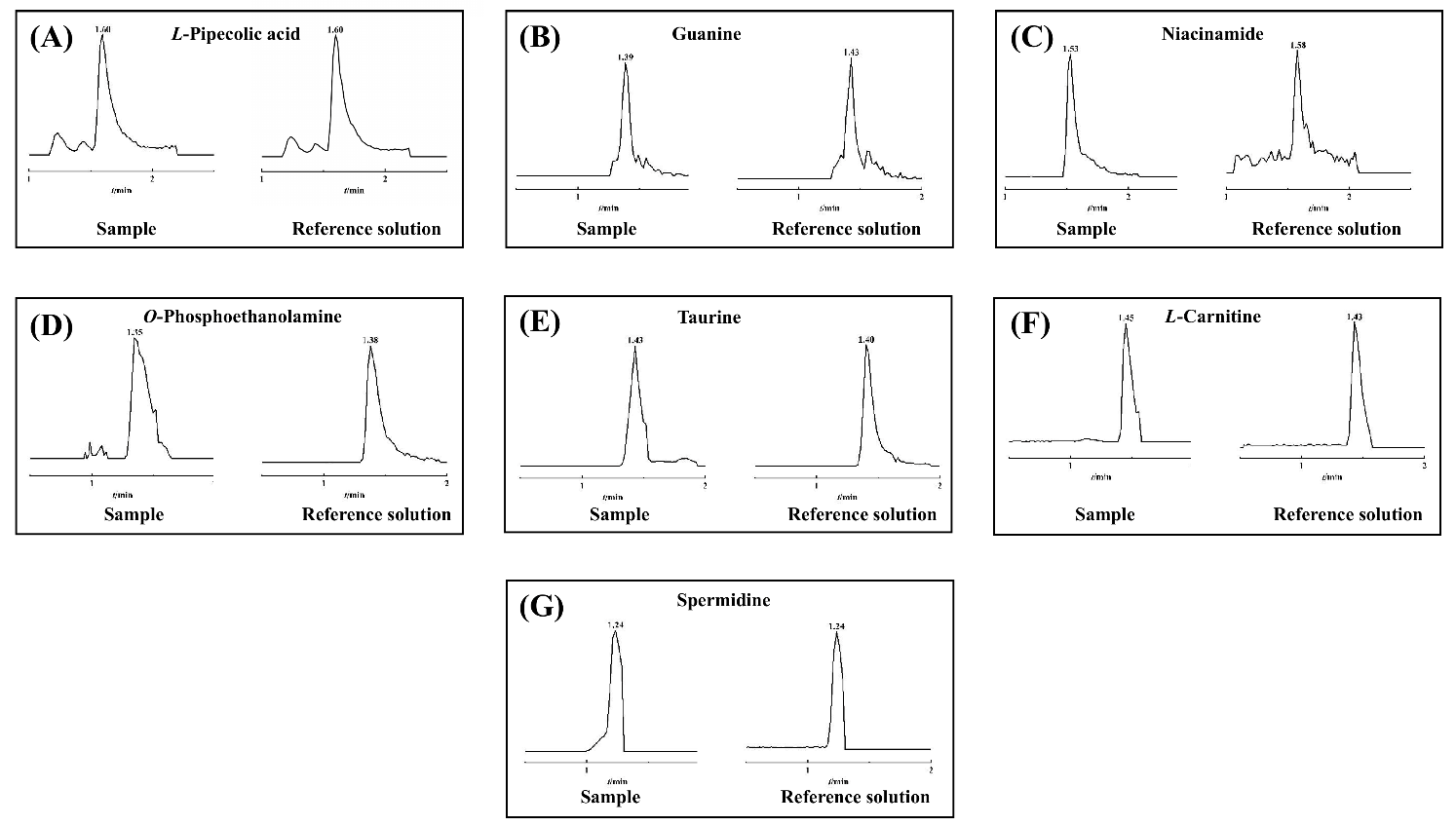


**Fig. 3S** Chromatograms of *L*-Pipecolic acid (A), Guanine (B), Niacinamide (C), *O*-Phosphoethanolamine (D), Taurine (E), *L*-Carnitine (F), Spermidine (G) plasma samples and reference solution.


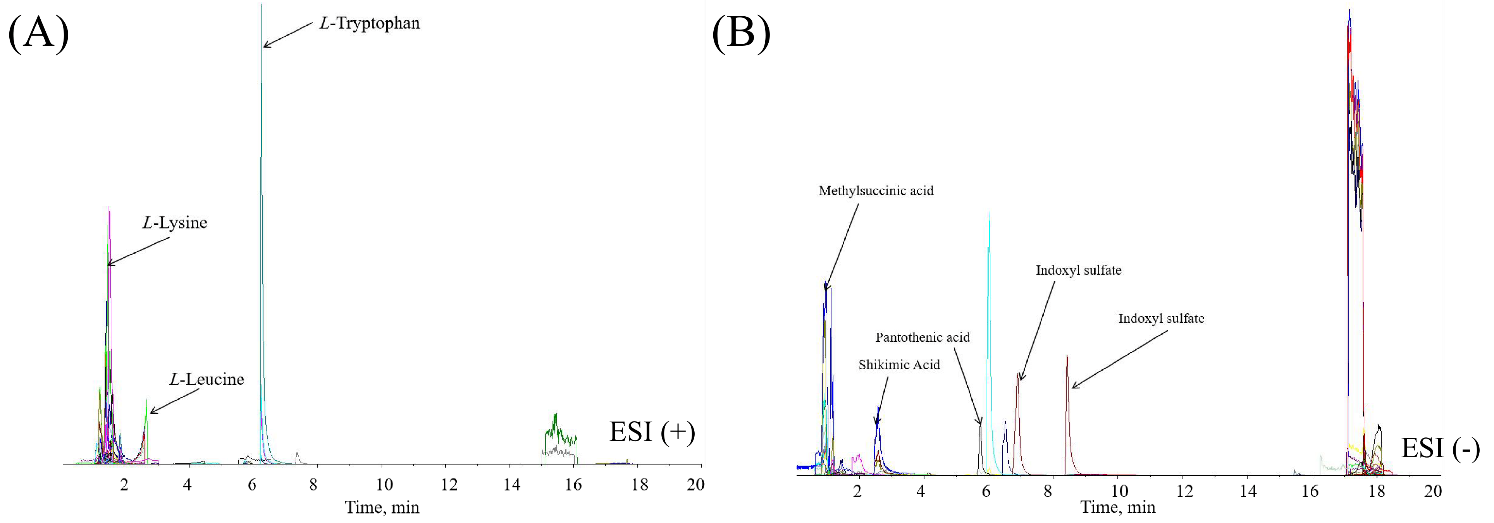


**Fig. 4S** Total Extracted Ion Chromatograms (EICs) of the Target Compound in Positive (A) and Negative (B) Ion Modes.
